# Obesity and its impact on female reproductive health: unraveling the connections

**DOI:** 10.3389/fendo.2023.1326546

**Published:** 2024-01-09

**Authors:** Lei Zheng, Lixian Yang, Ziru Guo, Nan Yao, Shiyu Zhang, Pengpeng Pu

**Affiliations:** ^1^ Department of Breast Surgery, Xingtai City People’s Hospital, Xingtai, Hebei, China; ^2^ Department of General Surgery, Aerospace Center Hospital, Beijing, China

**Keywords:** obesity, female infertility, adipose tissue, hormonal profile, reproductive health

## Abstract

In the modern era, the escalating global prevalence of obesity has profound implications on female reproductive health. Obesity, transcending mere lifestyle choices, has evolved into a complex disorder affecting physiological and metabolic functions. Concurrently, female infertility is rising as a significant global health issue. Obesity, with its extensive systemic effects, is pinpointed as a major disruptor. The convergence of these health challenges reveals a multifaceted scenario: on one hand, obesity directly impacts female reproductive health, particularly in the context of conditions like polycystic ovary syndrome (PCOS) and menstrual disturbances; on the other, the psychosocial consequences of infertility might intensify weight-gain patterns, forming a challenging cycle. Additionally, the economic implications of treating obesity-related infertility are considerable. This review delves into the myriad ways obesity affects female reproductive health, drawing insights from epidemiological, clinical, and molecular studies. It explores the epidemiological relationship between obesity and PCOS, the influence of obesity on menstrual disturbances, and the broader impact of obesity on female infertility. Weight loss, through pharmacological interventions, surgical methods, or lifestyle adjustments, emerges as a promising strategy. Lastly, the efficacy of assisted reproductive technologies, such as IVF, is influenced by obesity, underscoring the importance of an optimal body mass index. The review also highlights the molecular and physiological mechanisms underlying the impact of obesity on female reproductive health, including the disruption of the hypothalamic-pituitary-ovary axis, altered adipokine secretion, and the role of chronic inflammation and oxidative stress.

## Introduction

In the contemporary epoch, the burgeoning prevalence of obesity has emerged as a global health conundrum, casting a shadow over myriad facets of human well-being (1).Defined primarily by an excessive accumulation of adipose tissue, obesity is no longer merely a reflection of lifestyle choices; it has metamorphosed into a multifaceted disorder with profound implications on physiological and metabolic functions (2, 3). According to the World Health Organization, in 2011, more than 1.6 billion adults were overweight, and 400 million were obese highlighting the severity of this global issue (4).

Alongside the rising obesity issue, female infertility, defined as the inability to attain clinical pregnancy following 12 months of consistent unprotected intercourse, is increasingly becoming a major global health concern. The intricate dance of hormones, cellular processes, and anatomical structures that underpin female fertility is susceptible to perturbations, and obesity, with its widespread systemic effects, has been identified as a significant disruptor (5). The intersection of these two health challenges paints a complex picture. On one hand, obesity, with its associated metabolic and endocrine aberrations, can directly impinge on the reproductive health of women (6, 7). On the other, the psychosocial ramifications of infertility can exacerbate lifestyle patterns conducive to weight gain, creating a vicious cycle that is challenging to disrupt. The significance of understanding this relationship is manifold. Beyond the immediate health implications for the affected individuals, there are broader societal and economic repercussions. Infertility can be a source of profound psychological distress, impacting relationships, mental health, and overall quality of life (8). Moreover, in many cultures, childbearing is intricately linked with societal roles and expectations, and infertility can lead to stigmatization and marginalization.

Furthermore, the economic burden associated with treating infertility, particularly in the context of obesity, is substantial (9). From diagnostic procedures to therapeutic interventions, the costs can be prohibitive, placing additional strain on already stretched healthcare systems. In this review, we endeavor to elucidate the multifarious ways in which obesity impinges upon female reproductive health. Drawing from a rich tapestry of epidemiological, clinical, and molecular studies, we aim to provide a comprehensive overview of this critical intersection of metabolic and reproductive health.

## Impact of obesity on polycystic ovary syndrome

The epidemiological relationship between obesity and polycystic ovary syndrome (PCOS) has been extensively researched (10–12). PCOS, a hormonal disorder with irregular menstrual cycles and hyperandrogenism, is often linked with metabolic issues like insulin resistance. The complex interaction between obesity and PCOS influences both its etiology and management. Visceral adiposity, more than subcutaneous fat, is notably associated with the hormonal imbalances in PCOS, impacting female fertility (13). Obesity, particularly central obesity, exacerbates the metabolic and reproductive abnormalities associated with PCOS (14). Women with PCOS and obesity are at a heightened risk for insulin resistance, hyperinsulinemia, and type 2 diabetes (15). The hyperinsulinemia, in turn, can lead to increased ovarian androgen production, further aggravating the symptoms of PCOS (15). Additionally, obesity influences the secretion of various adipokines, such as leptin and adiponectin, which are known to play significant roles in reproductive health. Elevated leptin levels, often found in obese individuals, can disrupt normal ovarian function and are associated with the pathophysiology of PCOS (16). Conversely, adiponectin, known for its anti-inflammatory and insulin-sensitizing properties, is typically reduced in obesity and may contribute to the reproductive dysfunctions seen in PCOS (17). This vicious cycle underscores the importance of weight management in women with PCOS. Furthermore, the prevalence of PCOS varies across different populations and is influenced by diagnostic criteria and study design. However, studies clearly indicate a higher prevalence of PCOS among overweight and obese individuals. A study highlighted a 28.3% prevalence of PCOS in overweight and obese women, emphasizing the strong association between these conditions. Notably, the PCOS patients, with an average age of 26 ± 7 years, were significantly younger than the nonhyperandrogenic controls, who had an average age of 32 ± 8 years (18). In women with PCOS, cardiovascular risk factors such as hypertension and dyslipidemia are intensified by obesity. A previous study reported an adverse cardiovascular risk profile in women aged 25-34 years old diagnosed with PCOS, with obesity playing a significant role in this risk elevation (19) (**Table 1**).

**Table 1:** The relevant studies linking the obesity for PCOS, menstrual disturbances and female infertility.

Study	Design	Samples, n	Findings related to this topic
Cena H, 2020 (10)	Review	NA	Expanding the treatment options available to PCOS patients can be achieved through the weight loss effects of GLP-1 RA, which presents a distinct opportunity.
Gu Y, 2022 (11)	Review	NA	Lifestyle changes can serve as a valuable guide in managing PCOS and other related endocrine disorders, making them an invaluable tool for intervention.
Glueck CJ, 2019	Review	NA	Obesity, especially visceral adiposity, worsens metabolic and reproductive outcomes in PCOS.
Sirmans SM, 2013 (12)	Review	NA	Losing weight can lead to improvements in menstrual irregularities, symptoms associated with excess androgens, and infertility.
Moran LJ, 2009 (14)	Guideline	NA	Lifestyle management is the primary therapy for metabolic complications in overweight and obese women with PCOS. It may also improve ovulatory function and increase pregnancy chances for those with reproductive issues.
Hart R, 2004 (15)	Review	NA	The clinical presentation of PCOS can vary, but typically includes disturbances in the menstrual cycle, hyperandrogenism, insulin resistance, and obesity.
Estienne A, 2019 (16)	Review	NA	Endocrine factors play a role in regulating the secretion of GnRH, gonadotropins, and steroids.
Messinis IE, 2015 (17)	Review	NA	The impact of obesity on reproduction in PCOS is multifaceted, with hyperandrogenism, increased luteinizing hormone (LH), and insulin resistance all playing crucial roles.
Alvarez-Blasco F, 2006 (18)	Cross-sectional study	113	Overweight and obese women in Spain have a high prevalence of PCOS, with 28.3% affected. This highlights the need for healthcare providers to routinely screen for PCOS in premenopausal women who are overweight or obese and seeking weight loss advice.
Lo JC, 2006 (19)	Case-control study	22070	Individuals diagnosed with PCOS were found to have a higher likelihood of obesity (BMI ≥ 30 kg/m2; OR=4.21, 3.96-4.47) compared to those without PCOS.
Stella Fielder, 2023 (20)	Review	NA	Obesity is increasingly becoming a public health issue and is linked to menstrual disorders such as heavy bleeding, irregular periods, painful periods, and endometrial problems.
Norman RJ, 1998 (21)	Review	NA	Being overweight can cause reproductive problems such as menstrual disorders, infertility, miscarriage, and poor pregnancy outcomes, as well as an increased risk of diabetes.
Blüher M, 2013 (22)	Review	NA	Obesity can lead to increased estrogen production due to the activity of aromatase in adipose tissues. This hormonal imbalance can cause menstrual disturbances.
Nelson LR, 2001 (23)	Review	NA	The expression of aromatase in adipose tissue, and potentially in the skin, is responsible for the peripheral synthesis of estrogen. This expression increases with both body weight and age.
Krentz AJ, 2012 (24)	Case-control study	713	Leptin showed a positive association, whereas adiponectin demonstrated an inverse relationship with the increasing number of PCOS phenotype features.
Wei S, 2009 (25)	Case-control study	726	Obese women were found to have a 2-fold greater odds of experiencing an irregular menstrual cycle, regardless of whether it was defined by BMI (OR=2.61; 95%CI=1.28-5.35), WC (OR=2.28; 95%CI=1.16-4.49), or WHR (OR=2.27; 95%CI=1.09-4.72) when compared to women of normal weight.
Al-Safi ZA, 2015 (26)	Review	NA	Vasomotor symptoms are more severe in obese women, which may be attributed to the insulating effects of adipose tissue.
Bray GA, 2006 (27)	Review	NA	The different components of the syndrome, such as insulin resistance, dyslipidemia, and hypertension, can affect reproductive health and menstrual regularity.
Carson SA, 2021 (28)	Review	NA	Fertility can be adversely affected by various lifestyle and environmental factors, such as smoking and obesity.
Broughton DE, 2017 (29)	Review	NA	Obese women may have reduced fertility and lower success rates during in vitro fertilization due to factors such as hormonal imbalances, insulin resistance, and inflammation.
Talmor A, 2015 (30)	Review	NA	Being obese can lead to reproductive issues such as anovulation, subfertility, and infertility. It also increases the risk of miscarriage and can result in poor pregnancy outcomes for both the mother and newborn.
Ennab F, 2023 (31)	Review	NA	The relationship between obesity and infertility is well-known, but the underlying mechanisms and optimal management approaches are still uncertain.
Chen CI, 2015 (32)	Retrospective study	422	The metabolic complications in women with PCOS may be influenced significantly by adipose tissue.
van der Steeg JW, 2008 (33)	Cohort study	3,029	In ovulatory women with subfertility, obesity has been linked to reduced pregnancy rates.
Poston L, 2016 (34)	Review	NA	Obese pregnant women face higher risks of early pregnancy loss, congenital fetal malformations, delivering large infants, premature birth (both spontaneous and medically indicated), stillbirth, gestational diabetes, and pre-eclampsia, with potential long-term health consequences postpartum.
Zain MM, 2008 (35)	Review	NA	Being obese can lead to anovulation and menstrual irregularities, lower chances of conception, and a decreased response to fertility treatments.
Abdessalem H, 2012 (36)	Case-control study	582	There is a negative correlation between fertility and obesity/overweight, but this relationship may be affected by other variables.
Pasquali R, 2003 (37)	Review	NA	Obesity, especially the abdominal type, can cause reproductive issues due to complex mechanisms that aren't fully understood. However, a key factor is likely the presence of functional hyperandrogenism and hyperinsulinemia, which often accompanies insulin resistance.

NA, not applicable; OR, odds ratio.

## Impact of obesity on menstrual disturbances

Obesity’s influence on menstrual disturbances is multifaceted (20, 21). The adipose tissue, abundant in obese individuals, plays a significant role in steroid metabolism, leading to increased estrogen production. This increase is largely due to the activity of aromatase in adipose tissues, which converts androgens to estrogens. The elevated estrogen levels associated with obesity can disrupt the hormonal balance, potentially contributing to menstrual disturbances (22, 23). Obesity also alters the levels of adipokines like leptin and adiponectin, which are crucial in regulating reproductive hormones and menstrual cycles. High leptin levels in obese women can lead to menstrual irregularities and anovulation (24). Elevated estrogen levels can disrupt the regular menstrual cycle, leading to early menarche in adolescents and potentially early menopause in older women (25). Research has delved into the intricate relationship between obesity and the menopausal transition, exploring the impact of obesity on the timing of menopause and its symptoms (26). Furthermore, the metabolic syndrome, which is closely associated with obesity, has been linked to menstrual disturbances. The syndrome’s components, including insulin resistance, dyslipidemia, and hypertension, can influence reproductive health and menstrual regularity (27) (**Table 1**).

## Impact of obesity on female infertility

Obesity’s influence on female infertility is a topic of significant concern in reproductive medicine (28–31). The altered levels of adipokines in obesity, particularly the decrease in adiponectin and increase in leptin, are implicated in the pathogenesis of infertility. These changes can affect ovarian function, disrupt the hormonal balance necessary for ovulation, and impair endometrial receptivity (32). Several studies have delved into the intricate relationship between obesity and female infertility. In a prospective cohort study of 3,029 subfertile couples, a linear decline in spontaneous pregnancy rates was observed with each increase in body mass index (BMI) over 29 kg/m^2, showing a 4% decrease in pregnancy rates per kg/m^2 increase in BMI (33). One study discussed the epidemiological aspects and health consequences of obesity-related infertility, highlighting that assisted reproductive technology does not provide a straightforward solution to obesity-related infertility, as a high BMI also reduces the success rates of these treatments (34). Another research underscored the impact of obesity on female fertility and fertility treatments, emphasizing that the treatment of obesity should be the initial aim in obese infertile women before embarking on fertility treatments (35). A case-control study including 582 women found a significant negative association between obesity and female infertility, with an odds ratio of 3.26 for women with a BMI over 30 kg/m^2 (36). Furthermore, a comprehensive review provided insights into the epidemiology and pathophysiology of obesity in women and its implications for fertility (37) (**Table 1**).

## Impact of age and obesity on female reproductive health

The interplay between age and obesity significantly influences female reproductive health. In women of reproductive age, obesity is associated with various reproductive challenges, including impaired ovulatory function, reduced implantation and pregnancy rates, and increased miscarriage rates. These issues become more pronounced with advancing age, particularly in women approaching the upper limits of their reproductive years. For instance, women aged 38 years and older with obesity experience sub-optimal reproductive performance, impacting fertilization rates, embryo development, and pregnancy outcomes (38, 39). Additionally, overweight and obesity in early adulthood are linked to an increased risk of menstrual irregularities and hypertension in pregnancy (40). This evidence highlights the compounded effects of age and obesity on female fertility, emphasizing the importance of addressing these factors in reproductive health management.

## Molecular and physiological mechanisms

**Figure 1:**
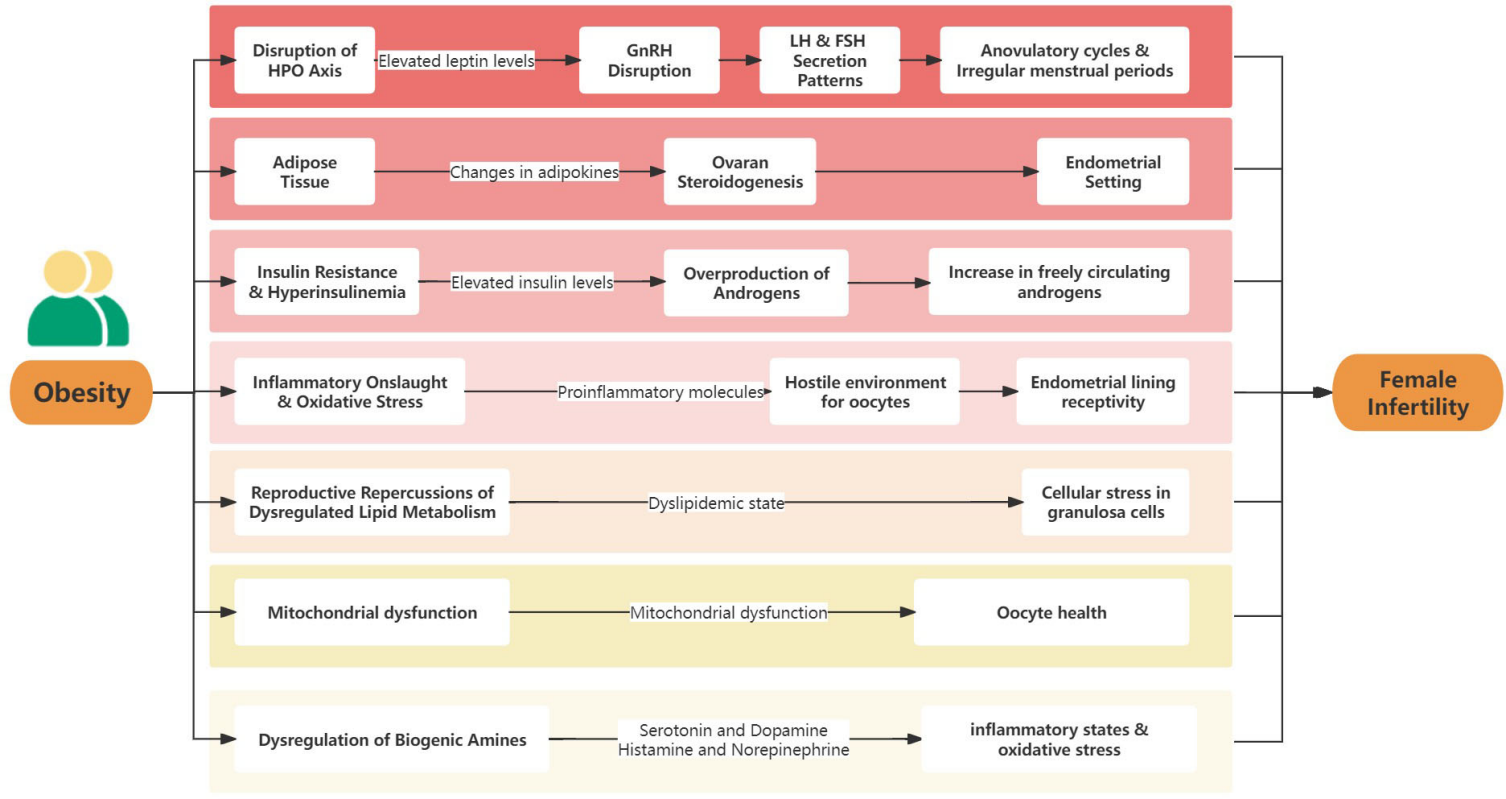
The mechanisms linking obesity to female infertility.

The complex relationship between obesity and female infertility is driven by numerous molecular and physiological mechanisms. Understanding these mechanisms is crucial to fully grasp this intricate association. At the cellular level, obesity induces a series of disruptions that significantly impact the reproductive system (**Figure 1**).

### Disruption of the hypothalamic-pituitary-ovary axis

The hypothalamic-pituitary-ovary (HPO) axis, which orchestrates a delicate balance of hormonal interactions, is fundamental to female reproductive physiology. It regulates the cyclical patterns of menstruation and the intricate process of ovulation (41). However, obesity disrupts this harmonious system, primarily through elevated leptin levels (42). Leptin, reactive oxygen species (ROS), and other adipokines are significantly altered in obesity, contributing to the dysregulation of the HPO axis. Leptin, an adipokine produced by adipose tissue, is significantly increased in obese individuals. This elevation in leptin can interfere with the rhythmic secretion of Gonadotropin-releasing hormone (GnRH) from the hypothalamus. Specifically, high levels of leptin are thought to disrupt the pulsatile nature of GnRH release. This disruption can lead to altered secretion patterns of luteinizing hormone (LH) and follicle-stimulating hormone (FSH), which are crucial for the normal menstrual cycle and ovulation (24). In obesity, the increased leptin levels may desensitize the GnRH neurons to leptin’s regulatory effects, leading to a dysregulation in the release of GnRH. This dysregulation can result in either an increase or decrease in the frequency and amplitude of LH and FSH pulses. The altered LH and FSH pulses can then impact follicular development, leading to menstrual irregularities and ovulatory dysfunction (43). Additionally, chemerin, another adipokine, is often elevated in obesity and metabolic syndromes, contributing to the disruption of normal reproductive functions. Chemerin has been implicated in the regulation of adipogenesis and inflammation, and its elevated levels in obesity are associated with insulin resistance and dysregulated lipid metabolism, further impacting the HPO axis (44). Adiponectin, typically decreased in obesity, plays a role in insulin sensitization and has anti-inflammatory properties. Its reduction in obesity can exacerbate the hormonal imbalances associated with reproductive dysfunctions (32). The decrease in adiponectin in obese individuals may contribute to insulin resistance, which can further impact the HPO axis by affecting the secretion and action of GnRH, LH, and FSH. The consequences are wide-ranging: from anovulatory cycles and irregular menstrual periods to a challenging fertility landscape. This hormonal imbalance not only diminishes natural conception chances but also poses challenges for assisted reproductive treatments.

### The endocrinological role of adipose tissue

Once regarded merely as a passive fat store, adipose tissue is now understood to be an active endocrine organ. In the context of obesity, the secretion patterns of adipokines, notably leptin and adiponectin, experience significant changes (45). Beyond their central role in metabolic homeostasis, these adipokines also intersect with reproductive functions. Imbalances in adipokine levels can affect ovarian steroidogenesis, resulting in a disrupted hormonal environment. This altered balance, particularly between estrogen and progesterone, can adversely affect the endometrial setting, rendering it less receptive to embryo implantation and early gestation (29).

### The dual threat of insulin resistance and hyperinsulinemia

Obesity’s distinct metabolic profile, marked by insulin resistance, has significant repercussions for reproductive health. Elevated insulin levels, which arise as a countermeasure to resistance, trigger a cascade of effects in the ovary. Specifically, they prompt the ovarian theca cells to overproduce androgens, leading to a hyperandrogenic state (46). This scenario, reminiscent of PCOS, is further exacerbated when insulin inhibits the liver’s synthesis of sex hormone-binding globulin (SHBG). The result is an increase in freely circulating androgens, which can interfere with ovulation, causing menstrual irregularities and reducing fertility potential (37).

### The inflammatory onslaught and oxidative stress

Obesity is frequently associated with chronic inflammation. In this condition, adipose tissue becomes a major producer of pro-inflammatory molecules, such as TNF-α and IL-6. Accompanying this rise in inflammation, a hallmark of obesity, is an increase in oxidative stress. This oxidative stress in obesity is characterized by an imbalance between the production of ROS and the body’s antioxidant defenses. The excess adipose tissue in obesity contributes to this imbalance, exacerbating inflammation and leading to a cycle of oxidative stress and further inflammatory response, which can disrupt metabolic homeostasis (47, 48). These combined factors create a hostile environment for oocytes, affecting their quality and viability. Furthermore, the inflammatory and oxidative conditions can negatively influence the endometrial lining, reducing its receptivity to embryo implantation and thereby presenting substantial challenges to successful conception (49).

### The reproductive repercussions of dysregulated lipid metabolism

The impact of obesity on lipid metabolism is significant. Elevated triglycerides, reduced high-density lipoprotein-cholesterol (HDL-C) levels, and a general dyslipidemic state have implications not only for cardiovascular health but also for reproductive outcomes (50). Lipotoxicity in ovarian granulosa cells, stemming from excessive lipid accumulation, can induce cellular stress through multiple interconnected pathways. The accumulation of lipids leads to increased ROS production, causing oxidative stress (51). Concurrently, this lipid overload disrupts endoplasmic reticulum (ER) function, triggering ER stress and the unfolded protein response, potentially leading to apoptosis if unresolved (52). Additionally, lipid accumulation can provoke an inflammatory response by stimulating pro-inflammatory cytokines, exacerbating cellular stress (53). This scenario is further complicated by impaired mitochondrial function, leading to decreased adenosine triphosphate (ATP) production and further ROS generation, contributing to the overall cellular stress and dysfunction in granulosa cells (54). Such stress can hinder their function, affecting oocyte maturation, follicular development, and overall reproductive capability (55).

### The mitochondrial malaise

Mitochondria, cellular powerhouses, play pivotal roles in a plethora of physiological processes, including oocyte maturation and embryonic development (56). However, obesity can lead to mitochondrial dysfunction. This dysfunction, marked by reduced ATP synthesis and increased ROS production, can adversely affect oocyte health, impacting both its cytoplasmic and nuclear maturity. Such challenges to mitochondrial function can limit the developmental potential of embryos, creating significant obstacles to successful conception and subsequent embryonic development (57).

In essence, the link between obesity and female infertility is supported by numerous molecular mechanisms, each adding to the diverse reproductive challenges encountered by obese women. As the obesity pandemic persists, a detailed understanding of these mechanisms becomes crucial in developing targeted therapeutic approaches to mitigate the reproductive issues caused by obesity.

### The role of biogenic amines in metabolic disturbances and reproductive health

Biogenic amines, such as serotonin, dopamine, histamine, and norepinephrine, are derived from amino acids and play critical roles in various physiological processes, including mood regulation, appetite control, and cardiovascular function. Recent research indicates a significant link between the dysregulation of these amines and metabolic disturbances associated with obesity, which in turn can have profound effects on female reproductive health (58). For instance, serotonin and dopamine have been implicated in the regulation of energy balance and appetite, processes often altered in obesity. Dysregulation in these neurotransmitters can contribute to the hormonal imbalances seen in obesity, indirectly affecting reproductive health (59). Additionally, histamine and norepinephrine are involved in inflammatory responses and stress regulation. The altered levels of these biogenic amines in obesity can exacerbate inflammatory states and oxidative stress, further impacting oocyte quality and endometrial receptivity (29). Alongside these biogenic amines, polyamines, including spermine, spermidine, and putrescine, are also integral to the discussion of metabolic and reproductive health. These polyamines are involved in cellular and genetic metabolism, aiding in transcription, translation, signaling, and post-translational modifications, which are crucial for maintaining cellular homeostasis and responding to metabolic challenges. Altered polyamine metabolism has been linked to various disease states, including metabolic disorders. Such dysregulation can have implications for reproductive health, as polyamines are known to be involved in cell growth and differentiation, processes that are essential for reproductive function (60–62).

## Weight loss as a therapeutic strategy for infertility in obese women

Weight loss emerges as a promising avenue for restoring reproductive health in obese women. A myriad of weight loss strategies, ranging from lifestyle modifications to medical interventions, have been explored for their efficacy in ameliorating obesity-induced infertility.

### Pharmacological interventions

Pharmacological interventions are key in addressing the reproductive challenges associated with obesity. Metformin, primarily an anti-diabetic drug, is widely recognized for its insulin-sensitizing properties, which have been beneficial in regulating menstrual cycles and improving ovulation rates in obese women (63). In addition to Metformin, glucagon-like peptide-1 (GLP-1) agonists, originally used for type 2 diabetes, have emerged as a promising option. These agonists have shown potential in improving insulin resistance linked with PCOS, commonly observed in obese women. By enhancing insulin sensitivity, GLP-1 agonists could potentially correct hormonal imbalances and improve fertility outcomes (10, 64). Moreover, antioxidant supplementation has been indicated as a potential strategy for improving reproductive outcomes in obese women. Studies suggest that antioxidants such as α-lipoic acid and myo-inositol may ameliorate oxidative stress in the oocyte environment, potentially contributing to improved fertility (65). Beyond these, the focus is also shifting towards drugs that target adipokines or their receptors. This includes exploring the therapeutic roles of agents that either enhance adiponectin activity or increase its levels, considering adiponectin’s crucial role in ovarian function and hormone production (66). Moreover, ongoing research is investigating molecules that counteract leptin resistance, a prevalent issue in obesity. Restoring leptin sensitivity through such interventions could play a significant role in addressing various reproductive dysfunctions linked to obesity (67).

### Surgical interventions

Surgical interventions, particularly bariatric surgery, have been increasingly recognized as a viable approach to address infertility challenges in obese women. Bariatric surgery, which is primarily designed to induce significant weight loss, has been shown to have a positive impact on fertility outcomes. One study found a notable improvement in obesity-related infertility following surgical intervention (29). Another research underscores the link between obesity and infertility, suggesting that weight loss induced by surgical means can be beneficial (35). A systematic review further emphasizes that weight loss in overweight or obese women can significantly enhance fertility treatment outcomes (68). Additionally, research provides insights into the positive outcomes of bariatric surgery in obese infertile women who wish to conceive, emphasizing that the success of these surgical interventions can vary based on factors such as age, the specific surgical procedure, co-morbidities, and BMI before surgery (69).

### Lifestyle modifications

Lifestyle modifications have emerged as a pivotal therapeutic strategy for addressing infertility in obese women. Comprehensive programs focusing on weight loss have demonstrated significant improvements in reproductive outcomes across various fertility treatments (70). Particularly in women with PCOS, a condition often associated with obesity and infertility, lifestyle changes have been shown to restore reproductive potential by enhancing insulin sensitivity and regulating luteinizing hormone levels (71). Furthermore, lifestyle interventions targeting central obesity and insulin resistance have been identified as crucial in managing PCOS-related infertility (72). A review on lifestyle factors in individuals seeking infertility treatment emphasized the potential benefits of intensive lifestyle modification programs in aiding weight management and improving fertility outcomes (73). Additionally, structured exercise training programs, when compared to specific dietary interventions, have also shown promise in treating obese PCOS patients with anovulatory infertility (74).

## Obesity and its implications on assisted reproductive technologies

Assisted reproductive technologies (ART), such as *in vitro* fertilization (IVF) and intracytoplasmic sperm injection (ICSI), have emerged as revolutionary tools in the realm of reproductive medicine, offering hope to countless couples facing infertility challenges. However, the interplay between obesity and the efficacy of these technologies has become a focal point of research and clinical concern. Obesity has been identified as a significant factor that negatively impacts various ART outcomes (75). Specifically, for women undergoing IVF, obesity has been associated with compromised pregnancy outcomes, underscoring the importance of optimal BMI in the context of ART (76). Interestingly, while obesity might diminish clinical pregnancy rates after IVF, its impact on ICSI cycles appears to be less pronounced, suggesting potential intrinsic sperm dysfunctions secondary to obesity might be circumvented in ICSI procedures (77).

## Conclusion

The rising global prevalence of obesity and its profound impact on female reproductive health has become a pressing concern. Obesity’s systemic effects, from hormonal imbalances to inflammation, disrupt the intricate processes of female fertility. Molecular mechanisms, including disruptions in the HPO axis and insulin resistance, further elucidate the challenges obese women face in reproductive health. Weight loss, through pharmacological interventions, surgery, or lifestyle changes, offers a promising solution. Additionally, the success of assisted reproductive technologies like IVF is influenced by obesity, highlighting the need for optimal body mass index. Addressing the intersection of obesity and infertility is crucial for improved reproductive outcomes worldwide.

## Author contributions

LZ: Writing – original draft. LY: Writing – review & editing. ZG: Writing – review & editing. NY: Visualization, Writing – review & editing. SZ: Methodology, Writing – review & editing. PP: Supervision, Writing – review & editing.
